# Development and validation of method for analysis of favipiravir and remdesivir in volumetric absorptive microsampling with ultra high-performance liquid chromatography–tandem mass spectrophotometry

**DOI:** 10.3389/fmed.2023.1022605

**Published:** 2023-05-05

**Authors:** Yahdiana Harahap, Roesytas Fitria Noer, Timbul Partogi H. Simorangkir

**Affiliations:** ^1^Faculty of Pharmacy, Universitas Indonesia, Depok, Indonesia; ^2^Pharmacy Study Program, Faculty of Medicine and Health Sciences, the Republic of Indonesia Defense University, Bogor, Indonesia

**Keywords:** favipiravir, remdesivir, VAMS, LC-MS/MS, COVID-19, acyclovir

## Abstract

Favipiravir and remdesivir are drugs to treat COVID-19. This study aims to find an optimum and validated method for simultaneous analysis of favipiravir and remdesivir in Volumetric Absorptive Microsampling (VAMS) by Ultra High-Performance Liquid Chromatography–Tandem Mass Spectrophotometry. The use of VAMS can be an advantage because the volume of blood is small and the sample preparation process is simple. Sample preparation was done by precipitation of protein using 500 μL of methanol. Analysis was carried out by ultra high-performance liquid chromatography–tandem mass spectrophotometry with ESI+ and MRM with m/z 157.9 > 112.92 for favipiravir, 603.09 > 200.005 for remdesivir, and at m/z 225.968 > 151.991 for acyclovir as the internal standard. The separation was carried out using an Acquity UPLC BEH C_18_ column (100 × 2.1 mm; 1.7 m), 0.2% formic acid—acetonitrile (50:50), flow rate was 0.15 mL/min, and column temperature was 50°C. The analytical method has been validated with the requirements issued by the Food and Drug Administration (2018) and European Medicine Agency (2011). The calibration range of favipiravir is 0.5–160 μg/mL and 0.002–8 μg/mL for remdesivir.

## 1. Introduction

Novel Coronavirus Disease-2019 (COVID-19) first appeared on 31 December 2019 in Wuhan City, China. On 31 December 2019, the Health Commission in Wuhan City reported a cluster of pneumonia diseases. This disease has mild symptoms, such as fever and dry cough and fatigue. Positive cases due to the COVID-19 outbreak are increasing in the world and in Indonesia. The number of cases in the world, as of 25th June 2021, was 179,686,071, while the number of deaths that occurred worldwide reached 3,899,172 cases with a mortality rate of 2.2% (1). Meanwhile, cases in Indonesia, as of 26th June 2021, have confirmed 2,093,962 cases with 56,729 deaths and a mortality rate of 2.7% (2). Until now, a specific drug for COVID-19 has not been found, but there are several drugs that have the potential to be used with emergency use of authorization. Some examples of potential drugs are lopinavir/ritonavir, favipiravir, remdesivir, chloroquine, and hydroxychloroquine (3).

Favipiravir and remdesivir belong to the class of antiviral drugs. Currently, favipiravir and remdesivir are licensed for use in the treatment of COVID-19 with an emergency use authorization. Favipiravir and remdesivir have been studied *in vitro* for inhibition of SARS-CoV-2. Favipiravir is effective *in vitro* to kill the SARS-CoV-2 virus with an effective concentration of 61.88 μM (4). While remdesivir, in a study conducted by Wang et al. in 2020, showed concentrations of 0.77 μM and 1.76 μM effective to kill 50% and 90% of Corona viruses including SARS-CoV-2 (5). A clinical study on remdesivir, conducted by Grein et al. in 2020, showed that 36 of 53 patients given remdesivir IV for 10 days showed improvement (6). It can be concluded that remdesivir can be used for the treatment of COVID-19. Currently, favipiravir and remdesivir are used in the treatment of COVID-19. According to the guideline in Indonesia, favipiravir is used for treatment the mild-to-severe COVID-19, while remdesivir is used to treat moderate to severe COVID-19.

The most fatal side effect of favipiravir is QT interval prolongation, while remdesivir is hepatotoxic in humans. QT interval prolongation can cause changes in heart rhythm that can cause arrhythmias. Remdesivir at a concentration of 6.026 ng/mL, *in vitro*, can cause toxic effects on human hepatocytes. This effect will result in an increase or decrease in enzyme levels in the liver so that the body’s metabolism will be disrupted (7). Meanwhile, favipiravir causes prolongation of the QT wave at a concentration of 157 μg/mL by blocking the hERG channel. Favipiravir has an effective concentration in COVID-19 disease of 40–80 μg/mL (8). Monitoring of favipiravir and remdesivir levels is necessary because favipiravir and remdesivir are new drugs that need to be re-examined regarding the safety and effectiveness of remdesivir and favipiravir in COVID-19 disease.

Analysis of favipiravir in biological matrices was carried out by Sağlam et al. and Morsy et al. in 2020 and remdesivir by Alvarez et al. and Avantaeo et al. in 2020 (9–12). Analysis of both favipiravir and remdesivir was performed in blood plasma. However, the use of plasma for analysis has several disadvantages. One of the disadvantage is the storage must be carried out at cold temperatures, thereby increasing costs in storage and shipping (13). One method other than fresh blood is the DBS or dried blood spot. The DBS biosampling method has several advantages, including only requiring a small sample and an easy collection process. In addition to its advantages, the DBS method has several disadvantages including the homogeneity of the sample depending on the level of red blood cells in the blood (hematocrit) and the volume taken cannot be maintained by the DBS sampling device. Thus, this can affect the drug concentration in the blood (13). The newest method is sampling by volumetric absorptive microsampling (VAMS). The highest advantage of this method is that the sampling volume is uniform and homogeneous because it does not depend on the amount of hematocrit present in the blood (14). The use of VAMS during the COVID-19 pandemic is recommended because the process is easy to use, and the volume taken is small so that it can be carried out by patient or volunteer without a medical background. The use of VAMS is easier than other micro sampling technique so that it can be studied through various media, such as short tutorials in the form of videos (15). Thus, the use of VAMS for monitoring drug levels in the blood can reduce the spread of the SARS-CoV-2 virus because the self-sampling process can be done.

Analysis of the similar drugs has been conducted with High-Performance Chromatography using both spectrofluorometric and UV detector. According to Imam et al. (2023), nirmatrelvir plus ritonavir, the similar drugs, can be analyzed using HPLC, but it needs more time to analyze and has higher lowest concentration range (16). Also, the analysis of remdesivir has been done by Batubara et al. (2023) using spectrofluorometric, but the remdesivir needs to be manipulated before analysis (17). Simultaneously, analysis of favipiravir and remdesivir has also already been done by Ramzy et al. (2022) by using spectrofluorometric method, but it also needs a lot of manipulation to overcome the overlap fluorescence spectra of both drugs (18). Therefore, by using LC-MS/MS, those challenge can be handled. Analysis using LC-MS/MS can be direct, not needing any additional steps for manipulation the drugs and also it can be more sensitive.

Method development and validation of favipiravir and remdesivir simultaneously with internal standard acyclovir by the VAMS biosampling and analyzed using Ultra High-Performance Liquid Chromatography–Tandem Mass Spectrophotometry has never been done before. Simultaneous analysis is expected to save time and chemicals used. Analysis using LC-MS/MS more often uses stable isotope labeling (SIL) because the ionization process is similar but the price of SIL is more expensive than analog compounds. In this study, acyclovir was used as the internal standard based on the consideration that acyclovir is an analog compound of remdesivir and favipiravir. Acyclovir is a base analog compound that has physicochemical properties similar to favipiravir and remdesivir. Therefore, this study needs to be carried out to obtain an analytical method that can be used later for monitoring blood levels of favipiravir and remdesivir.

The aim of this study is to develop a simple and fast way to analyze favipiravir and remdesivir in VAMS that can be used for pharmacokinetic study or drug monitoring. The novelty of this method lies on the sampling method, using the volumetric absorptive micro samplings (VAMS) and the simultaneous analysis. Sampling with VAMS can be beneficial in the pandemic situation because the patient can do the self-sampling with a guidance of healthcare professionals through video call or video and the simultaneous analysis can be more economical. First, we can save more reagents if we do the simultaneous analysis rather than one method only, and second, we can save more time because the analysis will happen faster than one method only. Another reason is that the method can cover more patient in the same condition, in this case COVID-19, rather than one drug one method only.

## 2. Result and discussion

### 2.1. Optimization of analysis conditions

The optimum condition for analysis favipiravir and remdesivir with ultra high-performance liquid chromatography with detection at m/z 157.9 > 112.92 for favipiravir and 603.09 > 200.005 for remdesivir and at m/z 225.968 > 151.991 for acyclovir as the internal standard using positive electrospray ionization (ESI) and multiple reaction monitoring (MRM). Those ions are used as quantifier and qualifier for the analytes.

The ratios of mobile phase that used for this optimization are 80:20 (v/v), 40:60 (v/v), 50:50 (v/v), and 20:80 (v/v) of 0.2% formic acid with acetonitrile, respectively. The optimum mobile phase is 0.2% formic acid with acetonitrile (50:50) in isocratic elution with flow rate of 0.15 mL/min. The temperature of the column is 50°C with a total running time of 3.5 min. Retention time of favipiravir is 1.72 min and remdesivir 2.82 min.

### 2.2. System suitability test

The system suitability test is carried out when the optimum analytical conditions have been obtained. This test was carried out as many as 5 replicas with the condition that %CV was less than 6.0% for retention time and peak area. The results of the system suitability test showed that the %CV area of favipiravir, remdesivir, and acyclovir, as internal standard, had a percentage of 1.21%, 3.43%, and 1.31%, respectively. Meanwhile, for the retention time of favipiravir, remdesivir, and acyclovir, the % CV was 0.49%, 0.16%, and 0.40%, respectively (Table 1). This shows that the analytical conditions used meet the requirements and the system works well. It shows that the analysis was accurate and precise.

**Table 1:** Data of system suitability test.

Data number	Area (μV/s)			Retention time (mins)		
	FPV	RDV	ACY	FPV	RDV	ACY
1	852586.00	1406588.00	1957764.00	1.72	2.82	1.37
2	847795.00	1486593.00	2017894.00	1.71	2.81	1.37
3	840923.00	1467673.00	2022353.00	1.73	2.82	1.36
4	863258.00	1497769.00	2003596.00	1.73	2.82	1.36
5	865340.00	1546604.00	2012484.00	1.72	2.82	1.36
Average	853980.40	1481045.40	2002818.20	1.72	2.82	1.36
SD	10317.63	50821.03	26138.10	0.01	0.00	0.01
%CV	1.21	3.43	1.31	0.49	0.16	0.40

### 2.3. Optimization of sample preparation

The optimum sample preparation for analysis of favipiravir and remdesivir with VAMS biosampling are initially by 2 h of drying time of the blood and continue with added 500 μL of methanol as the precipitation agent. The optimum time for vortex and sonication is 30 s and 20 min, respectively.

Sample preparation steps are absorbing the whole blood containing favipiravir and remdesivir with VAMS and held for 2 s after the tip turns red. The dried tip was separated from VAMS and moved to a microtube, then 10 μL of 10 ug/mL acyclovir as internal standard and 500 μL of methanol as the precipitating agent was added. The microtube was shaken using a vortex for 30 s, then sonicated for 20 min, and centrifuged for 10 min at 10.000 rpm. The supernatant was evaporated under N_2_ gas flow for 30 min under the temperature of 40°C then reconstituted with 100 μL of mobile phase, vortexed for 30 s, sonicated for 2 min, and centrifuged for 10 min at 10.000 rpm. The 10 μL of supernatant was injected to LC-MS/MS.

### 2.4. Validation of method

#### 2.4.1. Lower limit of quantification

**Figure 1:**
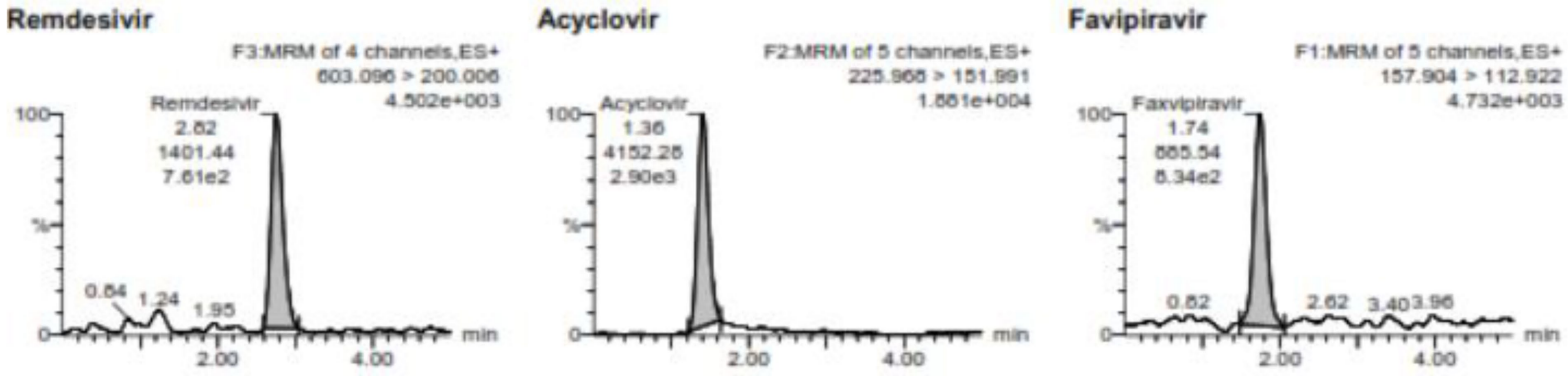
The chromatogram of the LLOQ for favipiravir and remdesivir including the internal standard of acyclovir.

Analysis of the lower limit of quantification (LLOQ) using five replicates of VAMS containing 500 ng/mL favipiravir and 2 ng/mL of remdesivir showed that %diff of favipiravir is −10.46% to 9.54% with %CV 9.42% and %diff of remdesivir is −11.19% to −0.6% with %CV 5.02%. The LLOQ of favipiravir was 500 ng/mL and remdesivir was 2 ng/mL. Figure 1 shows the chromatogram of LLOQ for both favipiravir and remdesivir.

#### 2.4.2. Calibration curve

The concentrations of favipiravir used for the calibration curve were 0.5, 5, 20, 40, 80, 100, and 160 μg/mL and for remdesivir were 2, 20, 500, 2,500, 5,000, 6,500, and 8,000 ng/mL including blank and zero. The calibration curves obtained are linear with correlation coefficient of >0.98 (18) (Table 2). The calibration curves were determined by calculating the ratio of analytes/IS then compare with analyte concentration by linear regression.

**Table 2:** Result of calibration of three consecutive days.

Replicates	Slope	Intercept	r
**Favipiravir**			
1	0.0003	0.5471	0.9979
2	0.0002	0.1100	0.9990
3	0.0002	0.1065	0.9995
**Remdesivir**			
1	0.028	1.387	0.9960
2	0.030	3.654	0.9970
3	0.020	1.288	0.9980

#### 2.4.3. Selectivity

**Figure 2:**
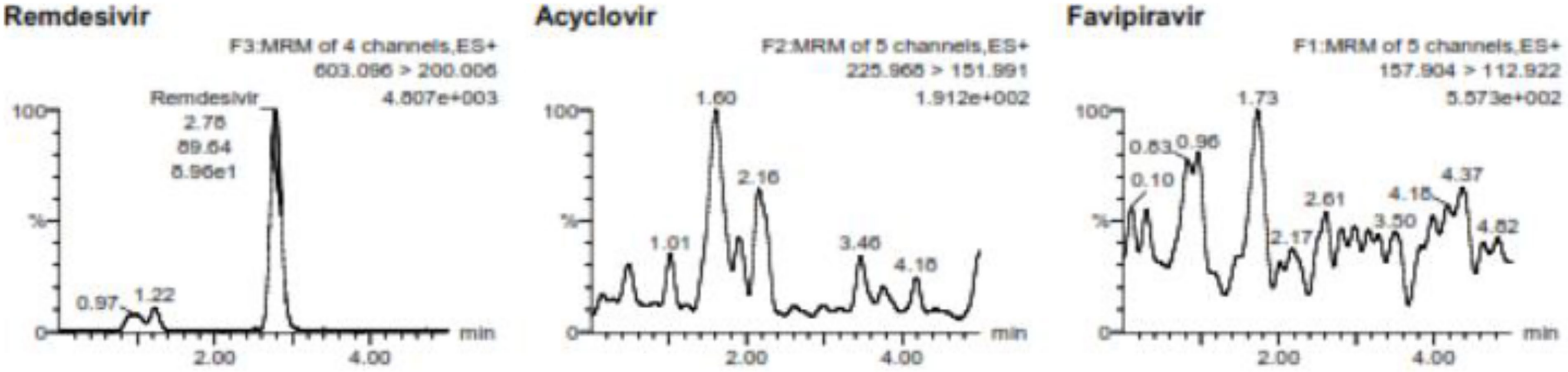
The blank chromatogram for favipiravir and remdesivir including the internal standard.

Analysis performed from 6 different blood sources resulted in interference values for favipiravir is 3.03%–5.50%, while for remdesivir is 8.60%–18.39%. Meanwhile, for the internal standard, acyclovir, the interference is 2.08%–4.03%. The amount of interference produced meets the acceptance criteria of FDA, so the method used is still selective for detecting favipiravir and remdesivir. Figure 2 shows the chromatogram of the blank from favipiravir and remdesivir.

#### 2.4.4. Accuracy and precision

**Figure 3:**
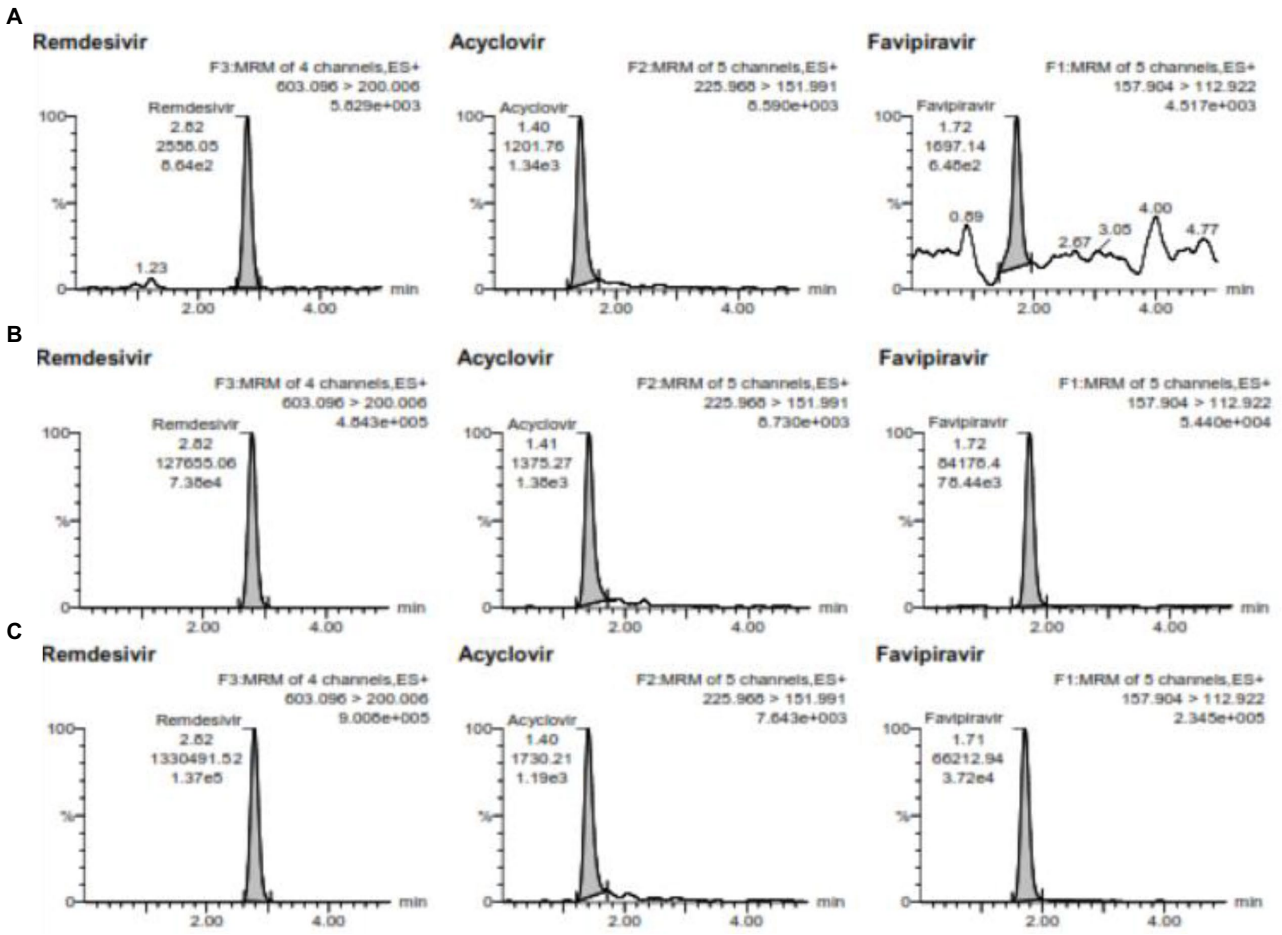
**(A)** is the chromatogram of QCL, **(B)** is the chromatogram QCM, and **(C)** is the chromatogram QCH for favipiravir and remdesivir including the internal standard of acyclovir.

Accuracy and precision conducted using 5 replicates in 4 different concentrations. The concentration used for favipiravir such as LLOQ is 0.5 μg/mL, QCL is 1.5 μg/mL, QCM is 7.2 μg/mL, and QCH is 120 μg/mL and for remdesivir such as LLOQ is 2 ng/mL, QCL is 6 ng/mL, QCM is 3,000 ng/mL, and QCH is 6,000 ng/mL at 3 consecutive days for between-run. The chromatogram for QCL, QCM, and QCH is shown in Figure 3. The data of accuracy and precision within- and between-runs are shown in Table 3.

**Table 3:** Within and between-run data.

Actual concentration (ng/mL)	Within-run			Between-run		
	Measured concentration (ng/mL)	%CV	%diff	Measured concentration (ng/mL)	%CV	%diff
**Favipiravir**						
500	511.89 ± 53.93	10.54%	−13.27% to 11.31%	519.23 ± 46.66	8.99%	−13.27% to 19.27%
1,500	1551.02 ± 76.72	4.95%	−3.18% to 11.08%	1493.63 ± 116.36	7.79%	−13.09% to 12.75%
7,200	7179.36 ± 569.61	7.93%	−7.31% to 12.27%	6972.56 ± 435.26	6.24%	−10.38% to 12.27%
120,000	117120.14 ± 5542.08	4.73%	−7.10% to 3.06%	112059.59 ± 7543.49	6.73%	−14.30% to 3.91%
**Remdesivir**						
2	1.98 ± 0.22	10.55%	−8.26% to 17.36%	1.96 ± 0.2	10.07%	−19.60% to 17.36%
6	6.27 ± 0.55	8.77%	−11.08% to 13.20%	6.05 ± 0.5	8.22%	−11.08% to 13.71%
3,000	2879.64 ± 266.60	9.26%	−11.63% to 10.44%	3023.29 ± 252.21	8.34%	−11.63% to 13.32%
6,000	6204.48 ± 485.50	7.82%	−3.56% to 14.41%	5820.52 ± 480.84	8.26%	−13.99% to 14.51%

#### 2.4.5. Recovery

The recovery of favipiravir with concentrations of QCL, QCM, and QCH obtained an average of 71.76%, 65.64%, and 75.11%, respectively with %CV of 0.93%, 4.57%, and 1.94%, respectively. Meanwhile, remdesivir at concentrations of QCL, QCM, and QCH obtained an average of 83.19%, 80.55%, and 83.38%, respectively, and %CV 0.11%, 0.57%, and 2.12%, respectively. According to the recovery requirements, %CV indicates that the extraction process is reproducible. ACY as an Internal standard was used and it has average recovery of QCL, QCM, and QCL concentrations are 71.48%, 64.54%, and 79.19%, respectively.

#### 2.4.6. Carry over

The results showed that there are carry over in both compounds, namely favipiravir and remdesivir, with a carryover percentage of 15.27% up to 17.21% for favipiravir and 13.67% up to 15.92% for remdesivir compounds. Meanwhile, at the internal standard, carry over is detected at 3.49% to 3.49%. 4.33%. The percentage of carryover is still within the acceptance limit so that it meets the requirements as FDA stated that the acceptance criteria of carryover should not exceed 20% of LLOQ.

#### 2.4.7. Matrix effect

The average matrix factor for favipiravir at the QCL concentration is 69.29% with a %CV of 12.88%, while for the QCH concentration, the average matrix factor was 88.24% with a %CV of 5.95%. Meanwhile, for the remdesivir compound, the average matrix factor at QCL concentration was 81.58% with %CV 13.07% and at QCH concentration is 81.99% with %CV of 5.05%. Acyclovir, as the internal standard, indicates the percentage of the matrix factor on average of 88.78% for the QCL concentration and 78.10% for the concentration of QCH with %CV of 5.07% and 8.34%, respectively, for the concentrations of QCL and QCH. The results showed that the average matrix factor values of favipiravir and remdesivir are below 100%. This shows that there is an ion suppression phenomenon that causes disruption of the compound ionization process because it suppresses the intensity of ionization (19).

To determine the influence of the matrix on the analysis, it is necessary to calculate the internal standard normalized matrix factor by comparing the analyte matrix factor and the internal standard matrix factor. The favipiravir compound has an internal standard normalized factor of 0.78 for the QCL concentration and 1.13 for the QCH concentration with %CV of 12.45% and 4.05%, respectively, for the concentration of QCL and QCH. Remdesivir had internal standardized factors of 0.92 and 1.05 for the concentrations of QCL and QCH, respectively, with %CV of 11.84% and 6.47%, respectively, for concentrations of QCL and QCH.

#### 2.4.8. Stability

##### 2.4.8.1. Stock solution stability

The stability of stock solutions is carried out in the short term, namely 0, 6, and 24 h, and the long term at 0 and 30 days. The short-term stability is carried out at room temperature and for a long term, it is carried out at −20°C. The test has requirements, namely accuracy with %diff not more than ±10% (20).

The results of the short-term stability test of favipiravir solution at short-term stability, are based on the conditions proposed by Merbel et al. (21), the stock solution of favipiravir is unstable at room temperature at 24 h. This could be due to the physicochemical properties of favipiravir, namely favipiravir including hygroscopic compounds (22). The results of the stability test of remdesivir stock solution showed that the stock solution of remdesivir is stable until 24 h.

Long-term storage of stock solutions is carried out in a refrigerator at 2°C–8°C for favipiravir and −20°C for remdesivir. The long-term stability results for favipiravir and acyclovir for storage at 2°C–8°C refrigerated are stable on day 30. Meanwhile, remdesivir on the 30th day showed unstable; therefore, the stock solution of remdesivir should be made fresh before the 30th day.

##### 2.4.8.2. Autosampler stability

Autosampler stability was assessed by immediately storing fresh extracted samples at QCL and QCH concentration in autosampler for 24 h. The result showed that favipiravir and remdesivir are stable at the autosampler for 24 h with %diff of favipiravir at 0 h are −14.16–3.21% with %CV of 5.04% (QCL) and 10.60% (QCH) and 24 h are −4.97%–3.29% with %CV of 3.39% (QCL) and 4.37% (QCH). While remdesivir has %diff of −13.41%–5.73% at 0 h with %CV of 8.42% (QCL) and 8.95% (QCH) also %diff of −14.48%–0.97% at 24 h with %CV of 5.05% (QCL) and 1.80% (QCH).

##### 2.4.8.3. Short-term and long-term stability in VAMS

Favipiravir and remdesivir are the drugs that are used for COVID-19. Therefore, analyzing remdesivir and favipiravir in a patient’s blood needs an inactivation process to prevent the spread of the virus at analysis period. The data at Table 4 show that favipiravir is unstable at 6 and 24 h. One of the reasons why favipiravir is unstable is that one of the chemical properties of favipiravir is hygroscopic, so it can absorb water from the air. The results of the stability test of remdesivir also unstable at 6 and 24 h. So, it can be concluded that favipiravir and remdesivir are unstable at 6 and 24 h at room temperature.

**Table 4:** Short-term stability test favipiravir and remdesivir in VAMS.

Hours	QCL			QCH		
	Measured conc. (average ± SD, ng/mL)	CV (%)	%diff	Measured conc. (average ± SD, ng/mL)	CV (%)	%diff
**Favipiravir (QCL = 1,500 ng/mL, QCH = 120,000 ng/mL)**						
0	1395.68 ± 70.29	5.04%	−11.05% to −1.84%	116582.78 ± 10145.53	8.70%	−8.68% to 6.85%
6	1.198 ± 95.20	7.94%	−27.37% to −15,74%	35733.62 ± 2079.92	5.83%	−23.73% to −68.56%
24	684.63 ± 378.08	55.22%	−79.51% to −29.10%	92704.30 ± 6413.92	6.92%	−27.53% to −16.98%
**Remdesivir (QCL = 6 ng/mL, QCH = 6,000 ng/mL)**						
0	5.81 ± 0.49	8.42%	−10.28% to 5.73%	5371.27 ± 271.25	5.05%	−13.41% to −5.27%
6	3.85 ± 0.1	2.64%	−36.25% to −33.96%	2117.99 ± 75.24	3.55%	−66.01% to 63.51%
24	3.33 ± 0.4	11.93%	−49.17% to −36.99%	1782.65 ± 467.17	26.21%	−78.80% to −63.52%

Long-term stability of favipiravir and remdesivir showed that favipiravir in the temperature of -20°C stable until day 14; meanwhile, remdesivir is not stable until day 7 (Table 5).

**Table 5:** Long-term stability test for favipiravir and remdesivir in VAMS.

Days	QCL			QCH		
	Measured conc. (average ± SD, ng/mL)	CV (%)	%diff	Measured conc. (average ± SD, ng/mL)	CV (%)	%diff
**Favipiravir (QCL = 1.500 ng/mL, QCH = 120.000 ng/mL)**						
0	1395.68 ± 70.29	5.04%	−11.05% to −1.84%	110123.22 ± 11929.45	10.83%	−14.77% to 3.21%
7	1558.05 ± 118.66	7.65%	−5.22% to 7.61%	121815.29 ± 1838.34	1.51%	0.15% to 3.17%
14	1448.24 ± 179.74	12.08%	−11.24% to 12.29%	104548.19 ± 964.53	0.92%	−13.65% to 12.05%
21	1222.11 ± 11.61	0.95%	−19.42% to −18.04%	94453.52 ± 1798.45	1.90%	−22.81% to −19.81%
30	854.08 ± 72.75	8.52%	−47.30% to −37.77%	62368.42 ± 1960.64	3.14%	−49.90% to −46.88%
**Remdesivir (QCL = 6 ng/mL, QCH = 6,000 ng/mL)**						
0	5.81 ± 0.59	8.42%	−10.28% to 5.73%	5371.27 ± 271.25	5.05%	−13.41% to −5.27%
7	4.85 ± 0.14	2.87%	−20.93% to −16.53%	3656.37 ± 184.32	5.04%	−42.15% to −36.00%
14	4.68 ± 0.38	8.04%	−27.40% to −15.17%	1317.80 ± 158.02	11.99%	−81.02% to −76.02%
21	4.67 ± 0.26	0.06%	−27.03% to −18.8%	1163.40 ± 125.14	10.76%	−82.56% to −78.41%
30	4.66 ± 0.28	6.04%	−27.37% to −18.12%	1140.26 ± 33.87	2.97%	−81.63% to −80.80%

## 3. Materials and methods

### 3.1. Material

Ultra high-performance chromatography tandem mass spectrophotometry consists of: Quaternary Solvent Manager Acquity UPLC H-Class (Waters, United States), Sample Manager FTN Acquity UPLC (Waters, United States), Nitrogen generator compressor (PEAK Scientific), Acquity Column UPLC BEH C18 (100 × 2.1 mm; 1.7 μm) (Waters, United States), mass analyzer in the form of a triple quadrupole Xevo TQD with ZsprayTM ionization source (Waters, Milford, United States); software for data processing (MassLynx Software, United States) and computers (Lenovo, China) pH meter (Eutech pH 510), analytical balance (AND GR-202, Japan), eluent and sample filter (Whatman), gas remover S60H (Elmasonic, Germany), Spectrafuge centrifuge (Labnet International, United States), ultrasonic stirrer (Elmasonic, Germany), −20°C freezer (Biomedical Labtech Deep Freezer), 2°C–8°C refrigerator (Samsung, Korea), vortex Maxi Mix II (Thermo Scientific, United States), Eppendorf micropipette (Socorex, Switzerland), TurboVap LV evaporator (Biotage, Sweden), vial autosampler and autosampler vial insert (Waters, United States), sample cup, blue tip, and yellow tip (Nesco, Indonesia), and other glassware (Pyrex, United States).

### 3.2. Reagents

Standard of favipiravir (Toronto Research Chemical, Canada), remdesivir (Cayman Chemical, America), and acyclovir (NADFC, Indonesia), whole blood (Indonesian Red Cross, Jakarta, Indonesia), and Volumetric Absorptive Microsampling (Neoteryx, Torrance, California, United States), formic acid HPLC Grade, ethanol HPLC Grade, acetonitrile HPLC Grade, and methanol HPLC Grade (Merck, Germany), ultrapure water treated with the Sartorius apparatus.

### 3.3. Method

#### 3.3.1. Optimization of analysis conditions

The optimized analysis conditions were detection on mass spectrophotometry, optimization of mobile phase combination, optimization of mobile phase composition, optimization of flow rate, and column temperature optimization. Detection in mass spectrophotometry was carried out by associating a solution of favipiravir, remdesivir, and acyclovir at a concentration of 1 ppm with reservoirs.

The combination of mobile phases was: 0.1% formic acid in water −0.1% formic acid in acetonitrile, formic acid 0.1% in water–acetonitrile, and 0.2% formic acid in water–acetonitrile with an isocratic composition of 20:80 (v/v). Mobile phase composition was 80:20 (v/v), 50:50 (v/v), 60:40 (v/v), and 20:80 (v/v). Combination of flow rates used after the selected combination and the composition of the mobile phase were 0.1; 0.15, and 0.2 mL/min, while the temperature combination column for optimization of column temperature was 40°C, 45°C, and 50°C.

#### 3.3.2. System suitability test

Solution containing favipiravir, remdesivir, and acyclovir in a concentration of 1 ppm, respectively, was injected into LC-MS/MS with an injection volume of 10 μL. Then, the analysis was carried out under optimum conditions for 5 injections. The conditions for the acceptance of the method are suitable for the desired compound analysis method is the correlation coefficient (%CV) obtained not more than 6%. The test was carried out before further analysis or validation on the same day (23).

#### 3.3.3. Optimization of sample preparation

The optimized sample preparation processes were tip drying time, volume extraction solution, vortex time, and sonication time. Variations of tip drying time for optimization were 1, 2, and 3 h. Volume of extracting solution, using methanol, varied with volumes of 500, 600, 800, and 1,000 μL. Meanwhile for variations, the vortex time used for optimization was 30 s and 1 and 2 min and sonication time was varied with 20, 30, and 40 min.

#### 3.3.4. Validation of method

##### 3.3.4.1. Lower limit of quantification

The LLOQ test was carried out by diluting the working standard solutions of favipiravir and remdesivir in the blood to a concentration of 500 ng/mL for favipiravir and 2 ng/mL for remdesivir and then prepared according to optimum conditions. This test was carried out with as many as 5 replicas. The LLOQ concentration meets the requirements if the analyte response produced is 5 times the blank response and the accuracy and precision value produced is 20%. If the test does not meet the requirements, then the test is carried out at a higher concentration (17).

##### 3.3.4.2. Calibration curve

Making a calibration curve using a minimum of 6 concentrations with a blank sample and zero sample (the blank sample plus the internal standard). The acceptance criteria is ±15% of the nominal concentration unless the LLOQ must be ±20%. In addition, at least 75% of the concentration in the calibration curve must meet these criteria (17). The area data from the calibration curve are recorded and the linear regression equation is calculated and then the correlation coefficient is obtained. The requirement of correlation coefficient is not less than 0.9800 (18).

##### 3.3.4.3. Selectivity

Selectivity was performed on six different matrix sources, in the form of whole blood, with LLOQ concentrations of favipiravir and remdesivir. The selectivity was carried out by two replicas and evaluated through interference in the blank sample. The criterion for acceptance of the selectivity test is the response of the peak area of not more than ±20% of the analyte LLOQ area and 5% of the internal standard area (18, 23).

##### 3.3.4.4. Accuracy and precision

Accuracy and precision tests were carried out within-run and between-run. The within-run accuracy and precision test were carried out on a single run, while the between-run test was carried out by means of 3 runs on at least 2 different days (European Medicines Agency, 2011). The concentrations used were 4 concentrations of 5 replicas per concentration for within-run analysis. The concentrations used were 0.5 μg/mL (LLOQ), 1.5 μg/mL (QCL), 72 μg/mL (QCM), and 120 μg/mL (QCH) for favipiravir and 2 ng/mL (LLOQ) and 6 ng/mL (QCL), 3,000 ng/mL (QCM), and 6,000 ng/mL (QCH) for remdesivir. The acceptance criterion for the accuracy test is ±15% of the nominal concentration unless the LLOQ must be ±20% of the nominal concentration. Meanwhile, the acceptance of the precision test is %CV not more than ±15% except for LLOQ with the requirement that the %CV is not more than 20% (18).

##### 3.3.4.5. Recovery

The recovery test was carried out by comparing the analyte content in the extracted matrix with the spiked blank with the analyte after extraction. The concentrations used were 1.5 μg/mL (QCL), 72 μg/mL (QCM), and 120 μg/mL (QCH) for favipiravir and 6 ng/mL (QCL), 3,000 ng/mL (QCM), and 6,000 ng/mL (QCH) for remdesivir. In the extraction process, 20 μL of acyclovir with concentration of 10 μg/mL was added. The solution that has been made, taken with VAMS 30 μL and then prepared using the sample preparation method.

The spiked blank was prepared by extracting the blank and then added favipiravir and remdesivir to the concentrations of QCL, QCM, and QCH and the internal standard acyclovir with a concentration of 10 μg/mL as much as 20 μL. The recovery test was carried out 3 times at each concentration.

##### 3.3.4.6. Carry over

The carry-over test was carried out by injecting a blank sample after LC-MS/MS was injected with the highest concentration, ULOQ. Favipiravir and remdesivir were diluted with whole blood to obtain a ULOQ concentration of 160 ug/mL for favipiravir and 8 μg/mL for remdesivir. The acceptance criteria of the carryover test is if the value of carryover is not more than 20% of the LLOQ area and not more than 5% of the internal standard area (18, 23).

##### 3.3.4.7. Matrix effect

The matrix effect was carried out by comparing the area of the matrix containing the analyte with the area of the standard analyte solution. This test uses 6 sources of blood, in this case whole blood obtained from individual donors. Favipiravir with concentrations of QCH and QCL (120 and 1.5 μg/mL) and remdesivir with concentrations of QCH and QCL (6,000 and 6 ng/mL) are added to the extracted blood under optimum conditions. The matrix effect test is carried out with 2 replicas. The acceptance criteria for the matrix effect test is %CV not more than 15% and the standard normalized matrix factor is in the range of 0.8–1.2 (23).

##### 3.3.4.8. Stability

###### 3.3.4.8.1. Stock solution

The stability of the stock solution was carried out in the long and short term. Short-term testing was carried out after storage of the solution for 0, 6, and 24 h at room temperature and long-term until 30 days in the refrigerator temperature (−20°C). The stock solutions that will be tested for stability test were stock solutions of favipiravir, remdesivir, and acyclovir with a concentration of 1 μg/mL. The acceptance criteria is the %diff value in the comparison of areas with a certain time to 0 h and day having a value not more than ±10% for favipiravir, remdesivir, and acyclovir stock solutions (20).

###### 3.3.4.8.2. Short-term and long-term stability

Short-term stability was achieved by diluting favipiravir and remdesivir with blood to concentrations of QCL and QCH. VAMS tips containing favipiravir and remdesivir were dried under N_2_ gas at 56°C for 30 min as the inactivation process and then prepared according to the selected method. The stability test was carried out with 3 replicas for each concentration and was carried out after storing favipiravir and remdesivir with VAMS for 0, 6, and 24 h at room temperature. For the long-term stability, the test was carried out with 3 replicas for each concentration and was carried out after storing favipiravir and remdesivir with VAMS for 0, 7, 14, 21, and 30 days at freezer temperature (−20°C). Acceptance criteria for short-term and long-term stability are accuracy and precision at each concentration not more than ±15% nominal concentration and %CV not more than 15% (18).

## 4. Conclusion

The developed analytical method is valid based on the Food and Drug Administration (2018) and the European Medicine Agency (2011) guidelines. The method is linear with concentration range of 0.5–160 μg/mL for favipiravir and 0.002–8 μg/mL for remdesivir. The method can be applied for quantification of favipiravir and remdesivir in VAMS for *in vivo* studies.

## Data availability statement

The original contributions presented in the study are included in the article/Supplementary material, further inquiries can be directed to the corresponding author.

## Ethics statement

This study uses strains obtained from Indonesian Red Cross, Jakarta, Indonesia. Ministry of Health of Indonesia did not require the study to be reviewed or approved by an ethics committee because the blood used is donor blood collected by the Indonesian Red Cross and has received approval to be used as a research sample, in accordance with the regulation of the Indonesian Minister of Health Number 91 year of 2015 about blood transfusion service standards.

## Author contributions

All authors listed have made a substantial, direct, and intellectual contribution to the work and approved it for publication.

## Funding

This study was financially supported by the Directorate of Research and Community Services (DRPM), Universitas Indonesia, Depok, Indonesia.

## Conflict of interest

The authors declare that the research was conducted in the absence of any commercial or financial relationships that could be construed as a potential conflict of interest.

## Publisher’s note

All claims expressed in this article are solely those of the authors and do not necessarily represent those of their affiliated organizations, or those of the publisher, the editors and the reviewers. Any product that may be evaluated in this article, or claim that may be made by its manufacturer, is not guaranteed or endorsed by the publisher.
