# Supplementary material for: Development and validation of method for analysis of favipiravir and remdesivir in volumetric absorptive microsampling with ultra high-performance liquid chromatography–tandem mass spectrophotometry
Source: Front Med (Lausanne). 2023 May 5;10:1022605. doi: 10.3389/fmed.2023.1022605 (PMC10203959; doi:10.3389/fmed.2023.1022605)
Supplement: Supplementary file 1 [file Data_Sheet_1.docx]

**Supporting Information**

**Figure S1** Spectrum and Chemical Structure of Favipiravir


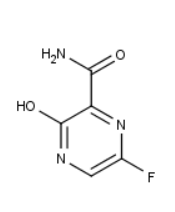
**
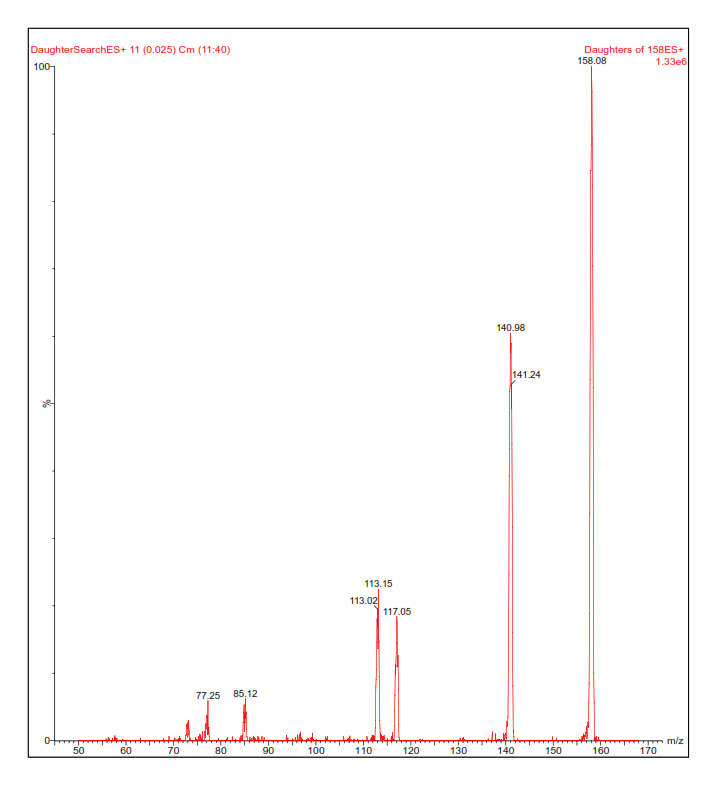
**

**Figure S2** Spectrum and Chemical Structure of Remdesivir


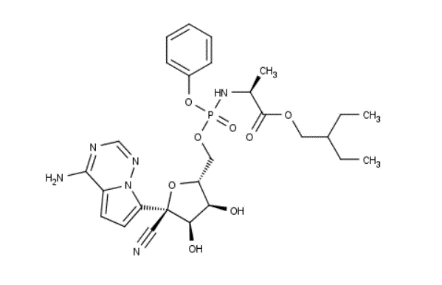
 **
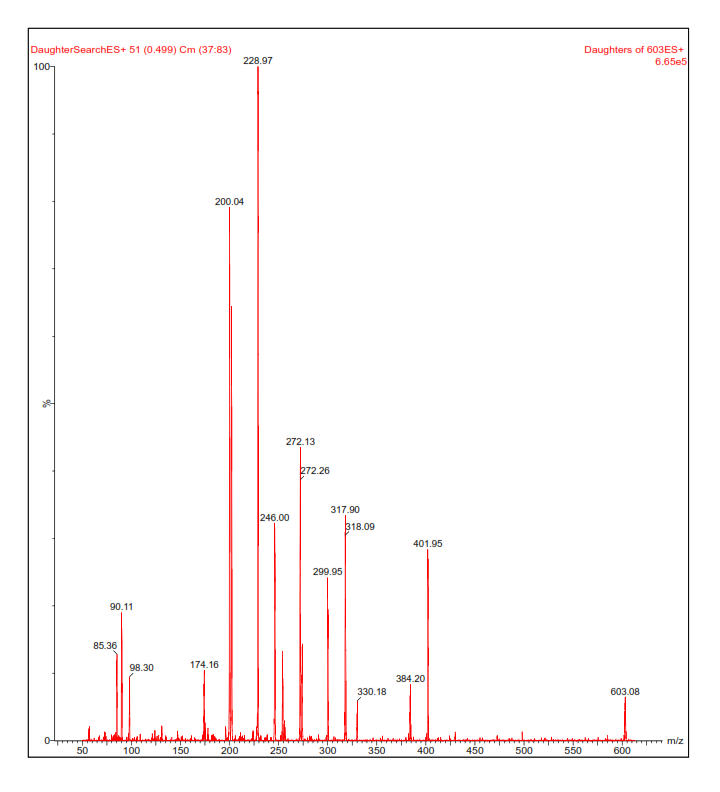
**

**Figure S3** Spectrum and Chemical Structure of Acyclovir as Internal Standard


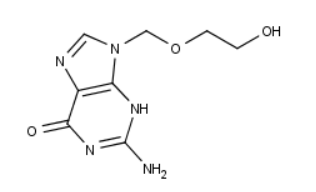

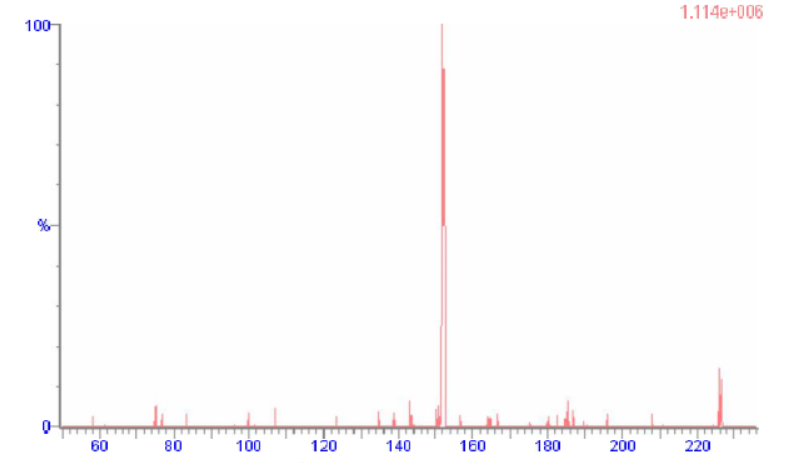


All the supporting informations are already included in the article
